# Therapeutic potential of PRL-3 targeting and clinical significance of *PRL-3 *genomic amplification in gastric cancer

**DOI:** 10.1186/1471-2407-11-122

**Published:** 2011-04-06

**Authors:** Akira Ooki, Keishi Yamashita, Shiro Kikuchi, Shinichi Sakuramoto, Natsuya Katada, Mina Waraya, Hiroshi Kawamata, Hiroshi Nishimiya, Kazunori Nakamura, Masahiko Watanabe

**Affiliations:** 1 Department of Surgery, Kitasato University Hospital, Kitasato 1-15-1, Sagamihara 228-8555, Kanagawa, Japan

**Keywords:** PRL-3, gastric cancer, genomic amplification, targeted therapy, lymph node

## Abstract

**Background:**

Phosphatase of regenerating liver-3 (PRL-3) has deserved attention as a crucial molecule in the multiple steps of metastasis. In the present study, we examined the mechanisms regulating PRL-3 expression, and assessed the clinical potential of PRL-3-targeted therapy in gastric cancer.

**Methods:**

PRL-3 genomic amplification was analyzed using quantitative-polymerase chain reaction and/or fluorescence in situ hybridization in 77 primary gastric tumors. The anticancer activity of PRL-3 inhibitor (1-4-bromo-2-benzylidene rhodanine) treatment was evaluated against cancer cells with different genetic and expression status.

**Results:**

PRL-3 genomic amplification was closely concordant with high level of its protein expression in cell lines, and was found in 20% (8/40) among human primary tumors with its expression, which were all stage III/IV disease (40%, 8/20), but in none (0/37) among those without expression. Additionally, PRL-3 genomic amplification was associated with metastatic lymph node status, leading to advanced stage and thereby poor outcomes in patients with lymph node metastasis (*P *= 0.021). PRL-3 small interfering RNA robustly repressed metastatic properties, including cell proliferation, invasion, and anchorage-independent colony formation. Although neither PRL-3 genomic amplification nor expression level was responsible for the sensitivity to PRL-3 inhibitor treatment, the inhibitor showed dose-dependent anticancer efficacy, and remarkably induced apoptosis on all the tested cell lines with PRL-3 expression.

**Conclusions:**

We have for the first time, demonstrated that PRL-3 genomic amplification is one of the predominant mechanisms inducing its expression, especially in more advanced stage, and that PRL-3-targeted therapy may have a great potential against gastric cancer with its expression.

## Background

Gastric cancer (GC) is the fourth most common cancer and the second leading cause of cancer-related death worldwide [1]. Recent improvements in diagnostic tools and methods have facilitated detection of early GC and thereby excellent long-term survival. However, patients with advanced disease at the time of diagnosis remain poor outcomes. Metastasis is a multistep process, involving local invasion, dissemination, and re-establishment into distant organs, and is the major determinant of the mortality [2]. Therefore, a better understanding of metastasis may open the way to a host of innovative therapeutic strategies in GC.

The protein tyrosine phosphatases (PTPs) form a large family of enzymes that serve as key regulatory components in signal transduction pathways [3]. The phosphatases of regenerating liver (PRL-1, -2, and -3), belonging to a small class of PTP superfamily, have a unique COOH-terminal prenylation motif, which critically affects their cellular localization and function [4]. PRL-3 was firstly identified to be specifically over-expressed in liver metastases derived from colorectal cancer [5], and subsequently its overexpression was documented in various tumor types, including GC [6]. PRL-3 can promote cancer invasion, migration, growth, and angiogenesis, through either dephosphorylation that is catalyzed by catalytic domain or localization to plasma membrane directed by COOH-terminal prenylation motif [7-9]. Thus, PRL-3 has deserved attention as a crucial molecule in the multiple steps of metastasis and therefore as a new therapeutic target. On the other hand, the mechanisms inducing PRL-3 expression are not fully clarified. Amplification of genomic regions containing oncogenes is the major mechanism of its consequent overexpression and the cancer development, and therefore has importance for targeted therapies [10]. *PRL-3 *gene amplification partially accounts for the overexpression in colorectal cancer and esophageal cancer [5,11]. However, the relationship between genomic amplification and GC remains elusive in the both mechanistic and therapeutic points of view. In the present study, we examined the characteristics of *PRL-3 *genomic amplification in GC, and further assessed the clinical potential of PRL-3-targeted therapy.

## Methods

### Cell lines and Tissue Samples

The GC cell line MKN7 was kindly provided from the Cell Resource Center for Biomedical Research Institute of Development, Aging and Cancer, Tohoku University (Sendai, Japan). Seven other GC cell lines (GCIY, AZ521, KatoIII, SH10, H111, MKN74, and NUGC4) were purchased from RIKEN BioResource Center (Ibaraki, Japan). These cell lines cover the two main types of GC [12], intestinal type (MKN7, MKN74, AZ521, and H111 cells) and diffuse type (GCIY, KatoIII, SH10, and NUGC cells) [13-15]. MKN7, NUGC4, and AZ521 cells were established from lymph node metastasis (LNM), and MKN74 cells were from liver metastasis. KATOIII and GCIY cells were established from metastatic pleural effusion and ascites, respectively. H111 and SH10 cells were established from the xenotransplantation. Normal skeletal muscle C2C12 cells were purchased from DS Pharma Biomedical Co., Ltd (Osaka, Japan). AZ521 and C2C12 cells were grown in DMEM medium (GIBCO, Carlsbad, CA) supplemented with 10% fetal bovine serum (FBS). The other cells were grown in RPMI1640 medium (GIBCO) supplemented with 10% FBS. 1-4-bromo-2-benzylidene rhodanine was purchased from Calbiochem Corp (San Diego, CA), which was identified as a PRL-3 inhibitor through high throughput screening using chemical library of Korea Chemical Bank, and inhibited PRL-3 phosphatase activity [16]. Indeed, phosphorylation of KRT8, PRL-3-interacting protein, induced by catalytically inactive mutant of PRL-3, but not by wild type, was confirmed by PRL-3 inhibitor treatment in a dose-dependent manner [17]. Moreover, anticancer efficacy of PRL-3 inhibitor treatment also showed to be similar to that of siRNA treatment in esophageal cancer or colorectal cancer [11,17].

Out of 173 formalin-fixed, paraffin-embedded, tissue samples series where we previously assessed PRL-3 expression status using immunohistochemical staining (IHC) in GC [6], 77 matched pairs of primary tumor tissues and the corresponding normal mucosa tissues were randomly selected from patients with differential stages according to the 13^th ^edition of the Japanese Classification of Gastric Carcinoma (JCGC) [18]; 40 pairs with positive PRL-3 expression (10 patients in Stage I, 10 in II, 10 in III, and 10 in IV) and 37 pairs with negative expression (10 patients in stage I, 10 in II, 9 in III, and 8 in IV). All patients underwent gastrectomy according to the gastric cancer treatment guidelines in Japan [19], and histopathologic examinations were done according to the JCGC. The 6^th ^edition of the International Union Against Cancer (UICC)/TNM classification was also used [20]. Table 1 depicts the detailed information on 77 patients. All tissue samples were collected at the Kitasato University Hospital, and informed consent was obtained from all patients. The present study was approved by the Ethics Committee of the Kitasato University.

**Table 1:** Correlation between PRL-3 gene amplification and clinicopathological variables in 77 patients with gastric cancer

		PRL-3 gene amplification				
			
Variables	Total number	Negativity		Positivity		*p *value
				
		Number	(%)	Number	(%)	
PRL-3 expression						0.006
Negativity	37	37	(100)	0	(0)	
Positivity	40	32	(80)	8	(20)	
Age (years)						0.726
<60	34	30	(88)	4	(12)	
≥60	43	39	(91)	4	(9)	
Gender						0.710
Male	51	45	(88)	6	(12)	
Female	26	24	(92)	2	(8)	
Lymphatic permeation						0.343
Absence	15	15	(100)	0	(0)	
Presence	62	54	(87)	8	(13)	
Vascular permeation						0.263
Absence	25	24	(96)	1	(4)	
Presence	52	45	(87)	7	(13)	
Differentiation						0.134
Well and moderate	31	30	(97)	1	(3)	
Poor	46	39	(85)	7	(15)	
Depth of invasion						0.006*
T1 (m and sm)	15	15	(100)	0	(0)	
T2 (mp and ss)	35	33	(94)	2	(6)	
T3 (se)	19	16	(84)	3	(16)	
T4 (si)	8	5	(63)	3	(38)	
Lymph node metastasis						0.022
Absence	29	29	(100)	0	(0)	
Presence	48	40	(83)	8	(17)	
JCGC lymph node status^†^						0.004*
N0	29	29	(100)	0	(0)	
N1	21	20	(95)	1	(5)	
N2	20	14	(70)	6	(30)	
N3 and distant lymph nodes	7	6	(86)	1	(14)	
UICC lymph node status^‡^						0.002*
N0	29	29	(100)	0	(0)	
N1	18	17	(94)	1	(6)	
N2	16	13	(81)	3	(19)	
N3 and distant lymph nodes	14	10	(71)	4	(29)	
JCGC stage						0.005*
I (IA and IB)	20	20	(100)	0	(0)	
II	20	20	(100)	0	(0)	
III (IIIA and IIIB)	19	15	(79)	4	(21)	
IV	18	14	(78)	4	(22)	
UICC stage						0.003*
I (IA and IB)	21	21	(100)	0	(0)	
II	20	20	(100)	0	(0)	
III (IIIA and IIIB)	16	13	(81)	3	(19)	
IV	20	15	(75)	5	(25)	

### Fluorescence in situ hybridization analysis

Fluorescence in situ hybridization (FISH) analysis was performed, as described previously [11]. *PRL-3 *is located on chromosome 8q24.3 (GenBank accession number NT 000008.9), and the chromosome 8 centromeric probe was used to estimate the copy number. Because *PRL-3 *FISH scoring algorithms had not been standardized, the assessment was based on the criteria of *HER2 *[21]. For each sample, at least 60 cancer cells were scored. Positive *PRL-3 *genomic amplification was defined as a ratio of *PRL-3 *to chromosome 8 centromere more than 2.2, and negative was the ratio of less than 1.8. If the ratio of *PRL-3 *to chromosome 8 centromere was 1.8 to 2.2, additional cells were counted, and the ratio of more than 2.0 was finally considered as positive [21]. Polysomy was defined as the mean chromosome 8 centromeric signals more than 3.0 per nucleus [22].

### Quantitative-genomic PCR

Tissue sections from tumor and the corresponding normal mucosa, obtained at least 5 cm from the tumor edge, were sharply dissected on hematoxylin and eosin-stained slides, and genomic DNA was subsequently extracted using of a QIAamp DNA FFPE Kit (QIAGEN Sciences, Hilden). Quantitative-genomic polymerase chain reaction (Q-PCR) was performed to quantify *PRL-3 *gene copy numbers using iQ™ Supermix (Bio-Rad Laboratories, Hercules, CA) in triplicate on the iCycler iQ™ Real-Time PCR Detection system (Bio-Rad). To normalize *PRL-3 *gene copy number per cell, ADAM metallopeptidase domain 2 (*ADAM2*, NT 923907.1), located on chromosome 8p11.2, was used as an endogenous reference because that gene amplification is defined as a copy number increase of a restricted region of a chromosome arm [10]. ∆Ct values were calculated as Ct (*PRL-3*)-Ct (*ADAM2*) for each sample. Relative copy number was determined as 2^-^^∆∆^^Ct^, where ∆∆C_t _= ∆C_t _(tumor)-∆C_t _(corresponding normal) [23]. The increases of more than 2-fold relative to the corresponding normal were considered as genomic amplification. Additional file 1 depicts detailed PCR condition and sequences of primer and probe used in the present study.

### Western blotting

Whole cell lysates were extracted in RIPA buffer (Pierce, Rockford, IL) supplemented with 10 μL/mL Halt™ Protease Inhibitor Cocktail Kit (Pierce) and Halt™ Phosphatase Inhibitor Cocktail Kit (Pierce), and the protein were separated on NuPAGE^® ^4-12% Bis-Tris Gel (Invitrogen) according to the manufacturer's protocol. Both detection and quantification of the specific proteins were performed using ATTO Light Capture (ATTO Corporation, Tokyo, Japan). Two colorectal cancer cell lines DLD-1 and SW480 cells (RIKEN BioResource) were used as the low and high expression controls, respectively, as described previously [11].

PRL-3 mouse monoclonal antibody (R&D Systems, Minneapolis, MN) and β-actin mouse monoclonal antibody (Sigma, St. Louis, MO) were used as described previously [11].

### PRL-3 small interfering RNA transfection

Cells were transfected with 1 μmol/L Accell SMARTpool, siRNA-PRL-3 (Thermo Fisher Scientific, Lafayette, CO) mixed with Accell siRNA Delivery Media (Thermo Fisher Scientific) according to the Thermo Scientific Dharmacon^® ^Accell™ siRNA Delivery Protocol [24]. The Accell Non-targeting Pool (siRNA-ctr) and Accell siRNA Delivery Media alone were used as a control for non-sequence-specific effects and as a mock-treatment, respectively.

### Anchorage-independent colony formation assay

Anchorage-independent cell growth was analyzed by plating 0.36% top agarose (Bacto™ Agar, Becton, Dickinson and Company, Franklin Lakes, NJ) containing 1 × 10^5 ^cells on a surface of 0.72% bottom agarose in 6-well plates [11]. Cells were fed weekly by overlying fresh soft-agar solution, and colonies were photographed after 2 weeks of incubation. The 50% effective concentration (EC_50_) value of PRL-3 inhibitor treatment was calculated based on the measurement of colony count.

### Proliferation assay and invasion assay

The proliferation assay was performed using Premix WST-1 Cell Proliferation Assay System (Takara Bio, Tokyo). Cells (2 × 10^3^) were seeded in 96-well, and the proliferative activity was measured by absorbance at 450 nm on designated sampling days. The sensitivity to PRL-3 inhibitor on antiproliferation was determined using the 50% inhibitory concentration (IC_50_) value after treatment for 72 hours.

The invasion assay was performed in the 24-well BD BioCoat™ Matrigel™ Invasion Chamber (BD Biosciences Discovery Labware, Bedford, MA). Cells that had invaded through the membrane were counted in four separated fields per well. Both experiments were done in triplicate.

### Apoptosis Assays

Apoptosis assays were performed using Guava PCA System (Guava Technologies, Inc., Hayward, CA). Cells (2 × 10^5^) were treated with the PRL-3 inhibitor at the indicated concentration in medium supplemental with 1.0% FBS for 72 hours, then stained with Annexin V and 7-AAD (Guava Nexin Reagent). The experiment was done in triplicate and analyzed using CytoSoft 2.1.5 software (Guava Technologies).

### Statistical Analysis

Fisher's exact test, or the Mann-Whitney *U*-test was used to statistically analyze the relationship between *PRL-3 *gene amplification and clinicopathological variables. One-way analysis of variance (ANOVA) with post-hoc test was used to compare between three groups for siRNA treatment (siRNA-PRL-3, siRNA-ctr, and mock). Student *t *test was used to evaluate therapeutic effect for the individual concentrations of PRL-3 inhibitor, compared with 0 μmol/L of PRL-3 inhibitor. The Kaplan-Meier method was used to estimate cumulative survival rates, and differences in survival rates were assessed with the use of the log-rank test. All deaths of patients were cancer-related, and disease specific survival (DSS) was measured from the date of surgery to the date of death or the last follow-up. *P *< 0.05 was considered to indicate statistical significance. All statistical analyses were conducted with JMP 7.0 software (SAS Institute, Cary, NC).

## Results

### PRL-3 expression and genomic amplification in gastric cancer cell lines

**Figure 1:**
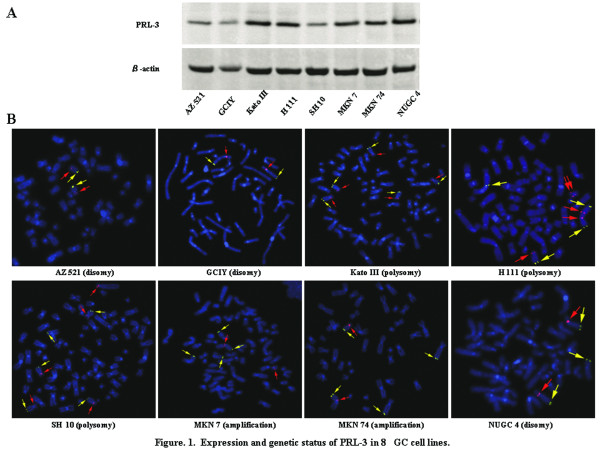
**Expression and genetic status of PRL-3 in 8GC cell lines**. (A) Expression level of PRL-3 by western blotting. (B) FISH analysis on metaphase of *PRL-3 *gene (green). The chromosome 8 centromeric probe (orange) was used to estimate the copy number.

Initially, PRL-3 expression status was evaluated using western blotting in 8 GC cell lines (Figure 1A). PRL-3 expression was observed at a detectable level in all the cell lines, among which 5 cell lines (KatoIII, H111, MKN7, MKN74, and NUGC4 cells) and 3 cell lines (GCIY, AZ521, and SH10 cells) exhibited high and relatively low expression, respectively. Subsequently, FISH analysis was performed to examine whether PRL-3 expression was caused through its genomic amplification (Figure 1B). Genomic amplification was obviously positive in 2 cell lines (MKN7 and MKN74 cells) and negative in 6 cell lines. 3 of the six were dysomic (AZ521, GCIY, and NUGC4 cells), and three were polysomic (KatoIII, SH10, and H111 cells). *PRL-3 *genomic amplification frequently occurred in the different regions from chromosome 8, so-called distributed insertions, on metaphase [10], and was concordant with its high expression.

### Characteristic of *PRL-3 *genomic amplification in human primary gastric cancers

**Figure 2:**
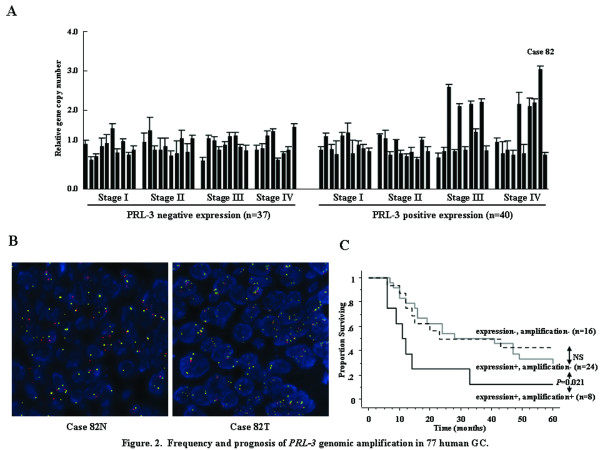
**Frequency and prognosis of *PRL-3 *genomic amplification in 77 human GC**. (A) Frequency of *PRL-3 *genomic amplification using Q-PCR in 77 human GC. Q-PCR was performed for both the 40 tumors with positive PRL-3 expression and 37 tumors with negative expression. To normalize *PRL-3 *gene copy number per cell, *ADAM2*, located on chromosome 8p11.2, was used as an endogenous reference. ∆Ct values were calculated as Ct (*PRL-3*)-Ct (*ADAM2*) for each sample. Relative copy number was determined as 2^-^^∆∆^^Ct^, where ∆∆C_t _= ∆C_t _(tumor)-∆C_t _(corresponding normal). (B) Representative FISH analysis of *PRL-3 *gene in primary tumor and corresponding normal (case 82). (C) Kaplan Meier curves of 5-year DSS according to the positivity or negativity of *PRL-3 *genomic amplification in the histologically node-positive patients. Error bars, standard deviation (SD).

In our previous study, PRL-3 expression was detected in 95 (55%) out of 173 primary GCs by IHC [6]. To explore the link between PRL-3 expression and its genomic amplification, Q-PCR was performed for both the 40 tumors with positive PRL-3 expression and 37 tumors with negative expression, which were randomly selected from differential stages in the 173 primary tumors. All the primary tumors without PRL-3 expression were not amplified, whereas 8 (20%) out of the 40 primary tumors with PRL-3 expression were amplified (Figure 2A). FISH analyses also confirmed obvious genomic amplification as the cancer-specific alteration (Figure 2B), and exhibited at nearly homogenous pattern in both the central area and invasive area within tumor. Subsequently, the relationship with clinicopathological factors was assessed for *PRL-3 *genomic amplification (Table 1), where it was significantly associated not only with its expression (*P *= 0.006), but also with depth of tumor invasion (*P *= 0.006), presence of LNM (*P *= 0.022), LNM status (*P *= 0.004 in JCGC, *P *= 0.002 in UICC), and stage (*P *= 0.005 in JCGC, *P *= 0.003 in UICC). Additionally, all the primary tumors with genomic amplification were stage III or IV disease (40%, 8/20). Moreover, the genomic amplification negatively affected the outcomes of the histologically node-positive patients (*P *= 0.021, Figure 2C), although PRL-3 expression did not in our and other previous reports [6,25].

### PRL-3 as a convergent therapeutic target

**Figure 3:**
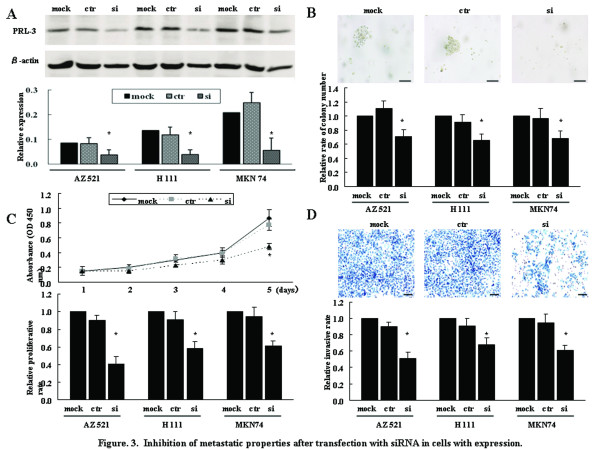
**Inhibition of metastatic properties after transfection with siRNA in cells with expression**. (A) Western blotting 72 hours after transfection. The western blotting was also quantified. mock, treatment with Accell siRNA Delivery Media alone; ctr, treatment with siRNA-ctr; si, treatment with siRNA-PRL-3. (B) Anchorage-independent colony formation assays. Representative pictures of colony formation on AZ521 cells were shown in top panel. With mock-treatment as 1.0, the relative rate of colony number was shown in bottom panel. Bars, 200 μm. (C) Proliferation assay. The proliferative activity of AZ521 cells on 1, 2, 3, 4, or 5 days after transfection (top panel) and the relative proliferative rate on 5 days after transfection (bottom panel) were shown. (D) Invasion assay. Cells were seeded on 4 days after transfection, and then incubated for 22 hours. Representative pictures of AZ521 cells (top panel) and the relative invasive rate (bottom panel) were shown. Bars, 200 μm; *, *P *< 0.05 by ANOVA with post-hoc test; error bars, SD.

In GC, the functional roles of PRL-3, including invasion and proliferation abilities, have been documented only in SGC7901 cells [25]. To confirm these metastatic properties using 3 cell lines with different PRL-3 expression and genetic status, knock-down of endogenous PRL-3 expression was performed using siRNA transfection; AZ521 cells (low expression and disomy), H111 cells (high expression and polysomy), MKN74 cells (high expression and genomic amplification). These cell lines were transfected with siRNA-PRL-3 or siRNA-ctr, and western blotting showed the decreased level of PRL-3 protein in siRNA-PRL-3 cells, but not siRNA-ctr cells, compared with mock-treatment cells (Figure 3A). One of the important characteristic of the metastatic phenotype is supposed as the ability for cancer cells to grow under anchorage-independent conditions [26], but the involvement in PRL-3 remains unknown in GC. All siRNA-PRL-3 cells showed the significantly decreased size and number of colonies, compared to siRNA-ctr cells or mock-treatment cells (Figure 3B). Moreover, in line with previous reports for other GC cell lines [25,27], we also confirmed that siRNA-PRL-3 cells showed the significantly less proliferative activity (Figure 3C) and invasive ability (Figure 3D).

### Therapeutic potential of PRL-3 inhibitor, 1-4-bromo-2-benzylidene rhodanine

**Figure 4:**
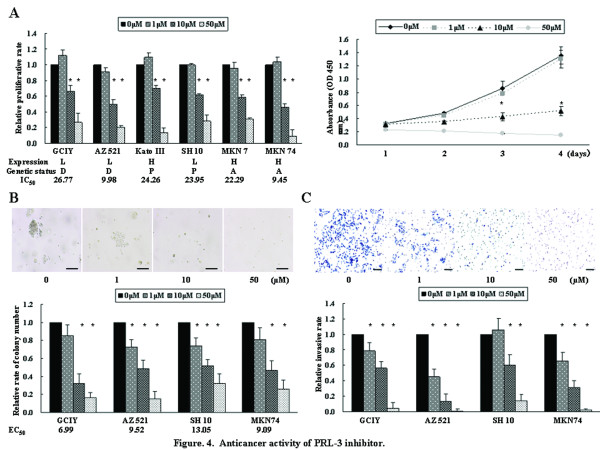
**Anticancer activity of PRL-3 inhibitor**. (A) Proliferation assay after treatment with PRL-3 inhibitor at concentrations ranging from 0 to 50 μmol/L against 6 cell lines with different PRL-3 expression and genetic status. With cells treated with chemical solution alone (0 μmol/L PRL-3 inhibitor) as 1.0, the relative proliferative rate on 5 days after treatment was shown in left panel. The proliferative activity of AZ521 cells on 1, 2, 3, or 4 days after treatment was shown in right panel. L, low expression; H, high expression; D, disomy; P, polysomy; A, amplification; IC_50_, the 50% inhibitory concentration. (B) Anchorage-independent colony formation assays. Representative pictures of colony formation on AZ521 cells (top panel) and the relative rate of colony number (bottom panel) were shown. Bars, 200 μm; EC_50_, the 50% effective concentration. (C) Invasion assay. Representative pictures (top panel) and the relative invasive rate (bottom panel) were shown. Bars, 200 μm; *, *P *< 0.05 by Student *t *test, compared with 0 μmol/L of PRL-3 inhibitor; error bars, SD.

**Figure 5:**
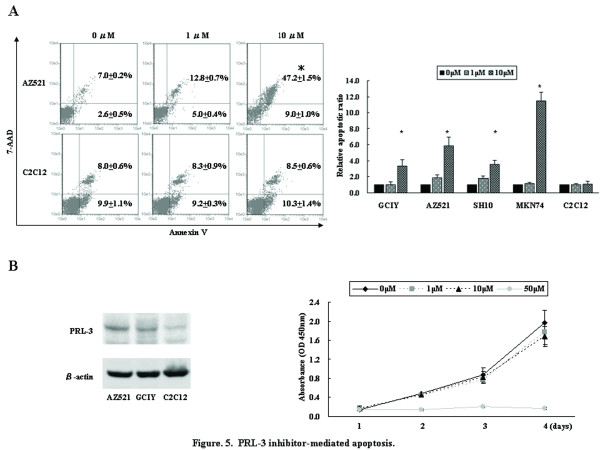
**PRL-3 inhibitor-mediated apoptosis**. (A) Apoptosis assay was performed 72 hours after treatment with PRL-3 inhibitor (0 to 10 μmol/L). Representative figures of apoptosis assay on AZ521 and C2C12 cells were shown in left panel, and the percentage and SD of early apoptosis (bottom right quadrant) and late apoptosis (top right quadrant) are shown in each panel. With cells treated with chemical solution alone (0 μmol/L PRL-3 inhibitor) as 1.0, the relative late apoptosis rate after treatment was shown in right panel. *, *P *< 0.05 by Student *t *test, compared with 0 μmol/L of PRL-3 inhibitor. (B) PRL-3 inhibitor treatment against normal skeletal muscle C2C12 cells. C2C12 cells exhibited lower expression level of PRL-3 than GC cells by western blotting. Proliferation assay after treatment with PRL-3 inhibitor was performed. Error bars, SD.

To assess the therapeutic potential and examine a landmark guiding the response to PRL-3-targeted therapy, we evaluated the anticancer activity of PRL-3 inhibitor, cell-permeable benzylidene rhodanine compound [16], against 6 cell lines with different PRL-3 expression and genetic status; GCIY and AZ521 cells (low expression and disomy), KatoIII cells (high expression and polysomy), SH10 cells (low expression and polysomy), MKN7 and MKN74 cells (high expression and genomic amplification). Cells were treated with PRL-3 inhibitor at concentrations ranging from 0 to 50 μmol/L. PRL-3 inhibitor showed dose- and time-dependent antiproliferative efficacy on all the tested cell lines, irrespective of different PRL-3 expression level and genetic status, and the IC_50 _values of GCIY, AZ521, KatoIII, SH10, MKN7, and MKN74 cells were 26.77, 9.98, 24.26, 23.95, 22.29, and 9.45 μmol/L, respectively (Figure 4A). AZ521 and MKN74 cells were more sensitive to PRL-3 inhibitor treatment than GCIY and MKN7 cells that were categorized as the identical groups in terms of expression and genetic status, respectively. Namely, genetic or expression status was not associated with sensitivity of GC cells against the PRL-3 inhibitor. Similar efficacy was shown in anchorage-independent colony formation, and the EC_50 _values of GCIY, AZ521, SH10, and MKN74 cells were 6.99, 9.52, 13.05, 9.09 μmol/L, respectively (Figure 4B). GCIY cells exhibited more sensitive inhibition in contrast with the anti-proliferation. Additionally, this inhibitor also robustly abrogated the invasive ability of GC cells (Figure 4C). To further characterize the anticancer efficacy of PRL-3 inhibitor treatment, apoptosis assay was performed (Figure 5A). Although 1 μmol/L of the inhibitor was insufficient to induce apoptosis beyond the baseline (0 μmol/L), 10 μmol/L of the inhibitor robustly caused the drastic apoptosis on all the tested cell lines, where there were the 3-fold and 11-fold increases beyond the baseline in GCIY and MKN74 cells, respectively. Thus, PRL-3 inhibitor repressed these metastatic properties on all the tested cell lines in dose-dependent manner, and neither expression level nor genetic status showed clear correlation with the sensitivity.

Finally, we assessed whether PRL-3 inhibitor induced cytotoxicity in normal skeletal muscle, where PRL-3 is predominantly expressed [28]. Both proliferation and apoptosis assays were performed using normal skeletal muscle C2C12 cells treated with the inhibitor, and showed that 10 μmol/L of the inhibitor failed to cause antiproliferative and apoptotic response on C2C12 in contrast with the efficacies on all the tested GC cell lines (Figure 5A and 5B).

## Discussion

As LNM is considered as an important prognostic factor for GC [29], research of the causative molecules reflecting LNM is a promising avenue to improve the outcomes. The close link of LNM with PRL-3 expression, therefore, has potential as a new therapeutic target [6,25]. However, the criteria for PRL-3-targeted therapy have not been established, and it is critical to clarify the characteristics of *PRL-3 *genomic amplification in the both mechanistic and therapeutic points of view, because of the major mechanism of its consequent expression and the cancer development [10]. In the present study, we offer the vital clues for the development of this therapeutic strategy against GC.

The relationship between PRL-3 expression and its genomic amplification have never been examined so far. *PRL-3 *genomic amplification was concordant with its expression status in cell lines, and was found in 20% (8/40) among human primary tumors with expression, which were all stage III or IV disease (40%, 8/20), but in none (0/37) among those without expression. Additionally, *PRL-3 *genomic amplification was associated with LNM status, leading to advanced stage and thereby poor outcomes in patients with LNM (*P *= 0.021). Thus, *PRL-3 *genomic amplification may be the more relevant alteration for LNM, and be one of the predominant mechanisms inducing its expression in the more advanced stage. However, most tumors expressing PRL-3 were not amplified, especially in the earler stage. In mouse embryonic fibroblast cells with wild type but not p53^-/-^, PRL-3 is induced in a p53-dependent manner [30]. The p53 mutation or loss of function, however, has been documented in all the GC cell lines used in the present study, except for NUGC4 cells (The TP53 Web Site, http://p53.free.fr/), indicating that there is other mechanism independently of p53 pathway. PRL-3 expression was reported to be regulated at transcriptional level by mitogenic cytokines, such as IL-6, IL-21, HGF or IGF-1 in myeloma cell lines [24], or as TGF-β in colon cancer cell lines [31]. Recently, PolyC-RNA-binding protein 1 (PCBP1) has been identified as a translational regulator of PRL-3 [32]. The alternative mechanisms at transcriptional or translational level may be involved to regulate PRL-3 expression.

We also confirmed that siRNA-mediated PRL-3 knockdown significantly repressed cell proliferation and invasion in line with previous reports for other GC cell lines [25,27], and furthermore for the first time revealed the reduced effect of colony formation under anchorage-independent conditions, supporting that PRL-3 may be attractive therapeutic target against GC. The success of molecular-targeted therapy depends on the identification of a landmark to select patients with more benefit from the therapy, such as activating mutation or gene amplification of EGFR in non-small cell lung cancer [33], and overexpression or gene amplification of HER2 in breast cancer [34]. Thus, genetic alteration or expression status is possible to be a landmark for molecular-targeted therapy, and it is indispensable to evaluate the anticancer activity of PRL-3 inhibitor treatment against cancer cells with different genetic and expression status. Although neither *PRL-3 *genomic amplification nor expression level was responsible for the sensitivity to PRL-3 inhibitor treatment, the inhibitor exhibited dose-dependent efficacy on all the tested cell lines with PRL-3 expression, and remarkably induced apoptosis in line with a previous report [35]. PRL-3 is not expressed in human adult stomach, and its expression is cancer-specific event [6,7]. Collectively, the presence of PRL-3 expression, but not expression level, may be sufficient to promote metastatic properties through activation of downstream signaling pathways, and the effective inhibition seems to have important implication for the success of this treatment. Combined with our previous findings demonstrating the high frequency of PRL-3 expression (55%, 95/173) [6], PRL-3-targeted therapy may be applicable for most patients with GC. The different sensitivity against PRL-3 targeting as shown in the present study may imply the additional alterations attenuating the dependence of PRL-3 signaling networks on cancer cells. Therefore, identification of molecules leading to the different sensitivity would shed light on the development of more sophisticated strategy.

Normal tissues with PRL-3 expression may be susceptible to adverse effects from the targeted therapy, especially in normal skeletal muscle and heart [28]. Interestingly, PRL-3 inhibitor treatment with the concentration of 10 μmol/L significantly repressed proliferation through apoptosis induction on all the tested GC cell lines, whereas did not on normal skeletal muscle C2C12 cells, implying that this concentration may act as an optimal dose of anticancer activity without severe effects against muscle cells, and normal cells may have a better apoptotic protective mechanism, even though PRL-3 is constitutively expressed [35]. As C2C12 cells might not be the best control because of relatively weak expression, further research will be necessary to validate our findings.

## Conclusions

We have for the first time demonstrated that *PRL-3 *genomic amplification is one of the predominant mechanisms inducing its expression, especially in more advanced stage, and that PRL-3-targeted therapy may have a great potential against gastric cancer with its expression.

## List of Abbreviations used

PRL-3: Phosphatase of regenerating liver-3; GC: gastric cancer; PTP: protein tyrosine phosphatase; JCGC: Japanese Classification of Gastric Carcinoma; UICC: the Union Internationale Contre Le Cancer; FISH: fluorescence in situ hybridization; Q-PCR: quantitative-genomic polymerase chain reaction; EC_50_: 50% effective concentration; IC_50_: 50% inhibitory concentration; ANOVA: analysis of variance; DSS: disease specific survival.

## Competing interests

The authors declare that they have no competing interests.

## Authors' contributions

AO conceived of the study, performed the study, drafted the manuscript and participated in coordination. KY participated in coordination and assisted in editing of manuscript. SK, SS, NK, MW, HK, and KN helped in the collection and analysis of clinical data. MW participated in coordination. All authors read and approved the final manuscript.

## Pre-publication history

The pre-publication history for this paper can be accessed here:

http://www.biomedcentral.com/1471-2407/11/122/prepub
